# Supplementary material for: The Evolution and Characterization of the RNA Interference Pathways in Lophotrochozoa
Source: Genome Biol Evol. 2024 May 7;16(5):evae098. doi: 10.1093/gbe/evae098 (PMC11114477; doi:10.1093/gbe/evae098)
Supplement: evae098_Supplementary_Data [file evae098_supplementary_data.zip › Supplementary_materials.zip!/selected_miRNAs/NoGe_selected_miRNAs/known_miRNA_families_pdf_files/NMRB01001262.1_Notospermus_geniculatus_strain_Ushimado_scaffold1262,_whole_genome_shotgun_sequence_26851.pdf]

Provisional ID	:	NMRB01001262.1_Notospermus_geniculatus_strain_Ushimado_scaffold1262,_whole_genome_shotgun_sequence_26851
Score total	:	107269.3
Score for star read(s)	:	3.9
Score for read counts	:	107260.7
Score for mfe	:	1.7
Score for randfold	:	0
Score for cons. seed	:	3
Total read count	:	210399
Mature read count	:	210206
Loop read count	:	0
Star read count	:	193

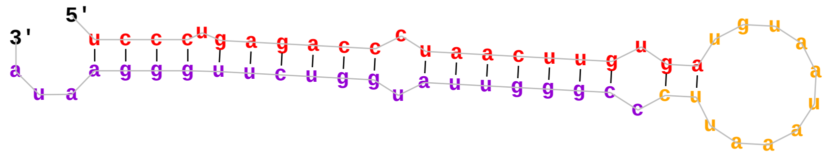

Genome coordinates: NMRB01001262.1 17772-17828

pre-miRNA sequence:
UCCCUGAGACCCUAACUUGUGAUCCCUGAGACCCUAACUUGUGAUGUAAUAAAUUCCCGGGUUAUGGUCUUGGGAAUA

Mature sequence: UCCCUGAGACCCUAACUUGUGA

Aligned mature sequence of the *N. geniculatus* pre-miRNA with the top 5 hits from the BLAST search against the pre-miRNA mirGeneDB set:

```
                              **********************
Nge_Mir-10-P3d                ucccugagacccuaacuuguga--
Tca-Mir-10-P3_5p              ucccugagacccuaacuugugaug
Tgu-Mir-10-P3d_5p             ucccugagacccuaacuuguga--
Xla-Mir-10-P3d3_5p            ucccugagacccuaacuuguga--
Xla-Mir-10-P3d4_5p            ucccugagacccuaacuuguga--
Xtr-Mir-10-P3d_5p             ucccugagacccuaacuuguga--
```
